# Sequestration and homing of bone marrow-derived lineage negative progenitor cells in the lung during pneumococcal pneumonia

**DOI:** 10.1186/1465-9921-9-25

**Published:** 2008-03-03

**Authors:** Hisashi Suzuki, James C Hogg, Stephan F van Eeden

**Affiliations:** 1 The James Hogg iCAPTURE Centre for Cardiovascular and Pulmonary Research, St. Paul's Hospital, University of British Columbia, Room 166, 1081 Burrard Street, Vancouver, British Columbia, V6Z 1Y6, Canada

## Abstract

**Background:**

Bone marrow (BM)-derived progenitor cells have been shown to have the potential to differentiate into a diversity of cell types involved in tissue repair. The characteristics of these progenitor cells in pneumonia lung is unknown. We have previously shown that *Streptococcus pneumoniae *induces a strong stimulus for the release of leukocytes from the BM and these leukocytes preferentially sequester in the lung capillaries. Here we report the behavior of BM-derived lineage negative progenitor cells (Lin- PCs) during pneumococcal pneumonia using quantum dots (QDs), nanocrystal fluorescent probes as a cell-tracking technique.

**Methods:**

Whole BM cells or purified Lin- PCs, harvested from C57/BL6 mice, were labeled with QDs and intravenously transfused into pneumonia mice infected by intratracheal instillation of *Streptococcus pneumoniae*. Saline was instilled for control. The recipients were sacrificed 2 and 24 hours following infusion and QD-positive cells retained in the circulation, BM and lungs were quantified.

**Results:**

Pneumonia prolonged the clearance of Lin- PCs from the circulation compared with control (21.7 ± 2.7% vs. 7.7 ± 0.9%, at 2 hours, *P *< 0.01), caused preferential sequestration of Lin- PCs in the lung microvessels (43.3 ± 8.6% vs. 11.2 ± 3.9%, at 2 hours, *P *< 0.05), and homing of these cells to both the lung (15.1 ± 3.6% vs. 2.4 ± 1.2%, at 24 hours, *P *< 0.05) and BM as compared to control (18.5 ± 0.8% vs. 9.5 ± 0.4%, at 24 hours, *P *< 0.01). Very few Lin- PCs migrated into air spaces.

**Conclusion:**

In this study, we demonstrated that BM-derived progenitor cells are preferentially sequestered and retained in pneumonic mouse lungs. These cells potentially contribute to the repair of damaged lung tissue.

## Background

*Streptococcus pneumoniae *is the most common cause of community acquired pneumonia and is one of the leading causes of death worldwide [1-3]. In addition to the local inflammatory response in the lung, pneumococcal pneumonia also induces a systemic immune response [4], which includes stimulation of the bone marrow (BM) with subsequent release of neutrophils and monocytes that participate in the inflammatory response in the lung. Studies from our laboratory demonstrated that pneumococcal pneumonia accelerates the transit time of both neutrophils and monocytes through the marrow and the release of these cells into the circulation [5,6]. A significant fraction of these newly released cells have immature characteristics and preferentially sequester in pneumonic regions of the lung [7].

The systemic inflammatory response induced by pneumonia is also characterized by an increase in circulating pro-inflammatory mediators [8-10], of which several (such as G-CSF and GM-CSF) are known to release hematopoietic stem cells from the BM [11,12]. Recent studies have shown that these BM-derived stem cells or progenitor cells have the ability to differentiate into cells that repopulate damaged tissues in different organs such as the heart [13], liver [14-16], brain [17] and lungs [18-21]. The majority of these studies have used models of toxic, traumatic or ischemic tissue injury, and showed engraftment of both type II [20,21] and type I [19] pneumocytes from BM-derived cells demonstrating the ability of these cells to participate in structural repair of the lung following injury. Therefore, we postulate that BM-derived progenitor cells will preferentially sequester in pneumonia-induced damaged lung tissue.

To test this hypothesis, we developed a novel labeling technique using quantum dots (QDs), which are fluorescent nanocrystals, to trace and quantify these progenitor cells in the lung. QDs have previously been used to label live cells for long-term multicolor *in vivo *imaging [22]. These nanocrystals are taken into cells by endocytotic pathways and the fluorescence of QDs persist intracellularly for more than a week [23]. Using this novel cell tracking technique, we measured clearance of infused whole BM cells (BMCs) and BM-derived lineage negative progenitor cells (Lin- PCs) from the circulation, their sequestration in the BM and lung, and their migration into airspaces in a mouse model of pneumonia.

## Methods

### Animals

Female C57BL/6J mice (10–12 weeks old) were used as donors and recipients. Mice were purchased from Jackson Laboratory (Bar Harbor, ME) and maintained on a standard laboratory diet and housed in a controlled environment with a 12-hour light/dark cycle in the animal care facility at Jack Bell Research Centre. All animal experiments were approved by the Animal Care Committee, University of British Columbia.

### Pneumonia model

For each experiment, *Streptococcus pneumoniae *(serotype 49619, ATCC, Rockville, MD) was used. A suspension of bacteria in saline at a concentration of 2.5 × 10^9 ^colony-forming units (CFU)/ml was prepared based on its optical density. Recipients were anesthetized with isoflurane and their tracheas were exposed by a small incision in the ventral portion of the neck. Bacterial suspension (250 μL/100 g body weight) was instilled into the trachea via a 28-gauge needle. An equal amount of sterile saline was used for control mice. The incision was sutured after the instillation. Following instillation, their weight was daily recorded and their behaviors, symptoms, and the condition of the wound were monitored twice a day until they were sacrificed.

### Isolation of BM cells

Femurs and tibias were removed from donors (uninfected, age-matched female C57BL/6J mice) and whole BM cells (BMCs) were obtained by flushing the marrow cavities with 10 ml phosphate-buffered saline (PBS) with a 25-gauge needle. The BM components were dispersed by repeated passage through a 10 ml syringe. The cells were washed twice with PBS+2% fetal bovine serum (FBS) and were filtered through 70 μm nylon mesh (BD Biosciences, San Jose, CA).

### Isolation of lineage negative progenitor cells

For the purified progenitor cell transfusion experiments, lineage negative progenitor cells (Lin- PCs) were separated from whole BMCs using a mouse progenitor cell enrichment kit (StemCell Technologies, Vancouver, Canada). Briefly, whole BMCs were incubated with assorted antibodies including rat anti-mouse CD5, CD45R, Mac-1, Gr-1, 7-4 and TER-119 that identify differentiated cells. After repeated washings to remove excess antibodies, the cells were incubated with magnetized microbeads that bind and eliminate the antigen-bound antibodies. Unbound Lin- PCs were purified by magnetic separation using AutoMacs (Miltenyi Biotec Inc, Auburn, CA) as a negative fraction under its "DEPLETES" program. The number of Lin- PCs was counted on the Cell-Dyn system (Abbott Laboratories, North Chicago, Il).

### Labeling of donor cells with QDs

BMCs and Lin- PCs obtained from donor mice were labeled with QDs (Qtracker 655 Cell Labeling Kit, Quantum Dot Corporation, Hayward, CA) by incubating with 10 nM QD-labeling solution at 37°C for 60 minutes. Cells were washed twice with PBS to remove the excess QDs.

### Transfusion of donor cells

Forty-eight hours following instillation of *S. pneumoniae *or control vehicle into the mouse lung, BMCs (1.0 × 10^6 ^cells/200 μl) or Lin- PCs (0.5 × 10^6 ^cells/200 μl) were transfused into the recipients via tail vein injection.

### Blood and tissue collection

Recipients were sacrificed at 2 or 24 hours after the cell transfusion. Blood was collected from the abdominal aorta with a 25-gauge needle. Femurs and tibias were obtained to isolate BMCs. Lungs were harvested and lung volume was measured by the water replacement method after inflating with 10% neutral-buffered formalin at 20 cmH_2_O. Lungs were then fixed in 10% formalin for more than 24 hours and each lung was cut into five slices for paraffin embedment.

### Flow cytometric analysis

Flow cytometric analysis was performed to determine the amount of QD-positive donor cells in recipients' peripheral blood and BM. Mononuclear cells (MNCs) were purified from whole blood by density gradient centrifugation with Histopaque-1077 (Sigma-Aldrich, St Louis, MO) at 400 g for 30 minutes. BMCs were isolated from femurs and tibias as described above. Both MNCs and BMCs were washed twice with PBS+2% FBS and were analyzed by a flow cytometer (Epics XL-MCL, Beckman Coulter Inc., Fullerton, CA) using the Summit software (Version 3.1, Cytomation Inc., Fort Collins, CO). Typically, 200,000 events for MNCs and 400,000 events for BMCs were acquired and the frequency of QD-positive cells was measured. The numbers of donor cells in circulation and BM were calculated as follows:

Number of QD-labeled donor cells in circulation = Fraction of donor cells in MNCs × fraction of MNCs × white blood cell count (/ml) × circulating blood volume (7 ml/100 g body weight)

Number of QD-labeled donor cells in BM = Fraction of donor cells in BM × total number of BMCs

The number of BMCs harvested from 2 femurs and 2 tibias was considered to represent 18.1% of total murine marrow [24]. The results were shown as percentages of total number of transfused donor cells.

### Histological analysis and detection of donor cells

Thin sections (5 μm) of lung tissue were prepared from paraffin-embedded blocks. Nuclei were stained with Hoechst 33342 (Invitrogen, Carlsbad, CA). Briefly, slides were incubated with diluted Hoechst 33342 solution (2 μg/ml) for 10 minutes at room temperature, followed by two washes with PBS for 10 minutes. The sections were coverslipped and examined using confocal microscopy (SP2 AOBS Confocal Microscope, Leica Microsystems GmbH, Germany) to detect QD-positive donor cells.

### Morphometric evaluation of QD-labeled donor cells in lung

The number of QD-labeled donor cells in recipient's lung was determined using a modification of the sequential level stereologic analysis [25]. A point-counting grid was placed over the images of lung slices taken at 4× magnification. The number of points falling on parenchyma was counted and its volume fraction (Vv) was estimated as follows:

$$\text{Vv parenchyma}=\frac{\text{Sum of the points on parenchyma}}{\text{Sum of the total points}}$$

For quantitation of QD-positive cells, 100 randomly selected fields of lung parenchyma were photodocumented from each mouse using confocal microscopy with a 63× objective lens. The Vv of QD-positive cells was calculated using a grid of 2025 (45 × 45) points superimposed onto the captured images. The density of the grid and number of fields counted were determined to maintain the coefficient of error below 0.2. The number of QD-labeled donor cells sequestered in the recipients' lung was calculated as follows:

Number of QD-labeled donor cells sequestered in lung = Lung volume × Vv lung parenchyma × Vv QD-positive cells × k^-1^

where k is the average volume of a mouse BMC as determined by measurement of 100 BMCs.

Localization of the QD-positive cells (either in lung parenchyma or in alveolar airspaces) was also recorded during the cell counting.

### Statistical analyses

All results were presented as mean ± standard error (SE). Statistical significance was evaluated using the unpaired Student's t-test for comparisons between two groups. Multiple comparisons were performed by one-way ANOVA and Tukey's post-hoc test. *P *< 0.05 was considered statistically significant. All statistical analyses were performed using SPSS software (Version 10.1, SPSS Inc., Chicago, IL).

## Results

### Evaluation of QDs in donor cells

**Figure 1:**
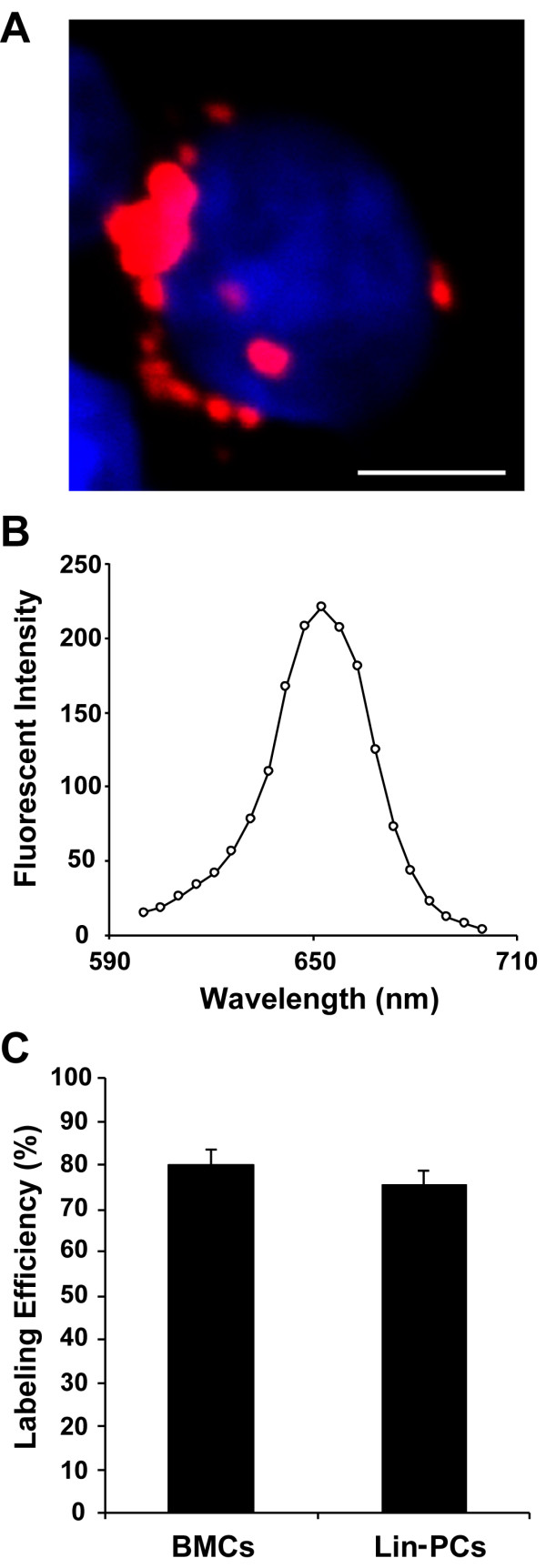
**Characteristics of labeled donor cells**. Isolated BM cells harvested from donor mice were labeled with QDs, which have an emission peak at 655 nm. A representative image illustrating a labeled cell emitting red fluorescence as observed under confocal microscopy is shown (A) and the red signal was confirmed as QDs by measuring their emission wavelength (B). Both the whole BM and Lin- progenitor cell populations were labeled with quantum dots and the labeling efficiency was 79.9 ± 3.4% and 75.5 ± 2.9%, respectively (C). Data is shown as mean ± SE, n = 5. Scale bar is 5 μm.

A representative image and an emission scan of QD-labeled donor cells are shown in Figure 1A and 1B. Fluorescent particles were detected within the cytoplasm of donor cells and the emission spectra of these particles peaked at 650 nm, thus confirming the identity and presence of QDs in the cells. The labeling efficiency of QDs in whole BMCs (79.9 ± 3.4%) and Lin- PCs (75.5 ± 2.9%) was measured by flow cytometry, as depicted in Figure 1C.

### Analysis of donor cells in the circulation

**Figure 2:**
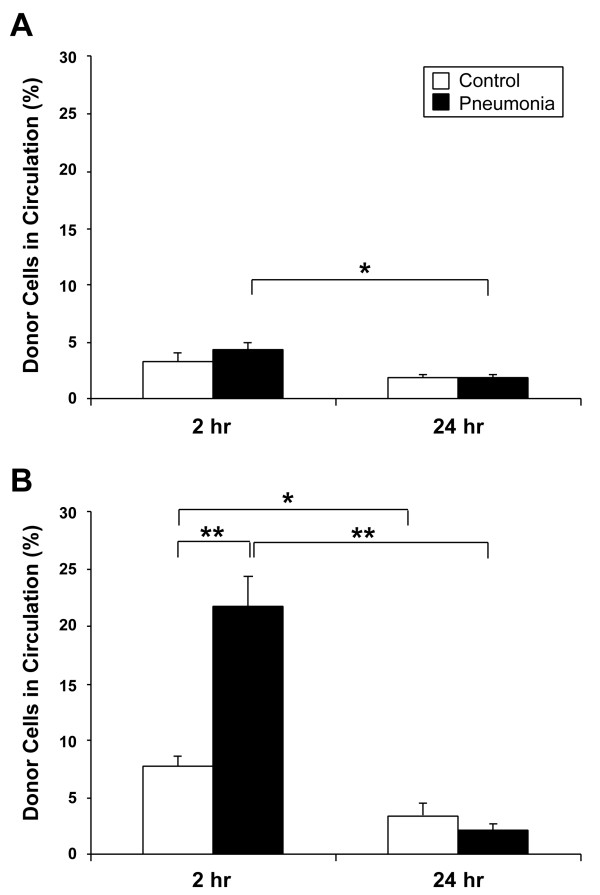
**Frequency of labeled donor cells in circulation**. Blood was collected from recipients which were sacrificed at 2 or 24 hours after cell transfusion. The frequency of labeled cells was measured by flow cytometry and the total number of labeled donor cells in recipients' circulation was calculated. The results are shown as percentages of total number of transfused donor cells. In the whole BM cell transfusion experiment (A), the proportion of donor cells at 2 hours post-transfusion was 3.3 ± 0.7% and 4.3 ± 0.7% in control and pneumonia groups, respectively (*P *= 0.34). However, the ratio of labeled cells in the pneumonia group significantly decreased to 1.8 ± 0.3% at 24 hours (*P *= 0.01). In the Lin- progenitor cell transfusion model (B), the proportion of labeled progenitor cells in the pneumonia group was significantly higher than control (21.7 ± 2.7% vs. 7.7 ± 0.9%, *P *= 0.008) at 2 hours. The percentage of donor cells in the circulation in both the control and pneumonia groups decreased after 24 hours and the difference between the two groups was no longer significant at 24 hours post-transfusion. Data is shown as mean ± SE; n = 6 (control group for each timepoint) and n = 7 (pneumonia group for each timepoint). *P < 0.05, **P < 0.01.

The number of QD-labeled donor cells, expressed as a ratio of total injected cells, detected in the circulation at 2 and 24 hours post-transfusion is shown in Figure 2.

In the whole BMC transfusion model (Figure 2A), the proportion of QD-labeled donor cells at 2 hours post-transfusion was 3.3 ± 0.7% and 4.3 ± 0.7% in control and pneumonia groups, respectively (*P *= 0.34). However, the ratio of labeled cells in the pneumonia group significantly decreased to 1.8 ± 0.3% at 24 hours, as compared to the previous timepoint (*P *= 0.01). In the Lin- PC transfusion model (Figure 2B), the amount of QD-labeled donor cells was significantly higher in the pneumonia group as compared to control (21.7 ± 2.7% vs. 7.7 ± 0.9%, *P *= 0.008) at 2 hours following transfusion. By 24 hours post-transfusion, the ratio of labeled cells significantly decreased to 3.4 ± 1.1% (*P *= 0.04) in the control group and to 2.1 ± 0.5% (*P *< 0.001) in the pneumonia group from 2 hours. However, at 24 hours there was no significant difference in the circulating Lin- PCs between the two groups (*P *= 0.29).

### Homing of donor cells to the BM

**Figure 3:**
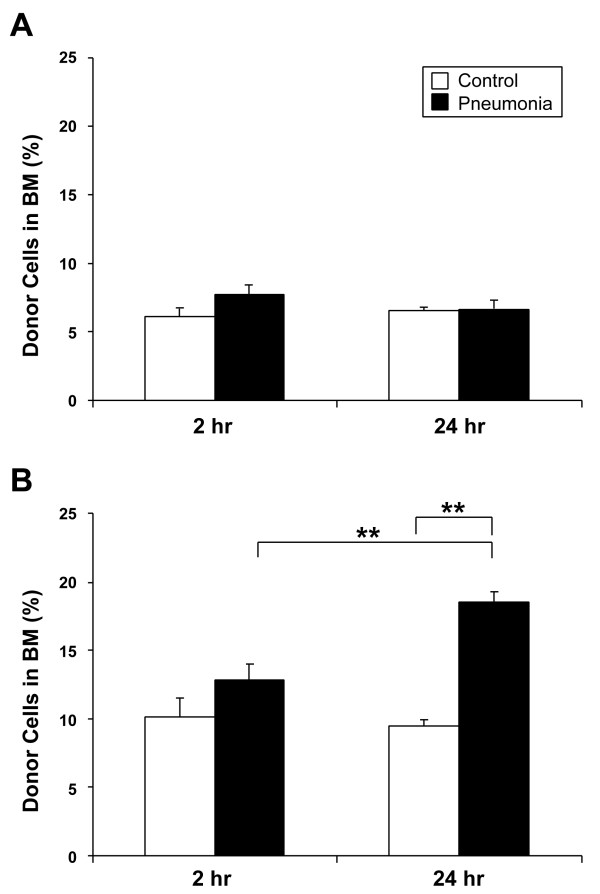
**Frequency of labeled donor cells in bone marrow**. BM cells were harvested from recipients which were sacrificed at 2 or 24 hours after the cell transfusion. The frequency of labeled cells was measured by flow cytometry and the total number of labeled donor cells in recipients' bone marrow was calculated. The results are shown as percentages of total number of transfused donor cells. In the whole BM cell transfusion experiment (A), there was an upward trend in the pneumonia group as compared to control (7.7 ± 0.6% vs. 6.1 ± 0.5%, *P *= 0.09) at 2 hours post-transfusion, although by 24 hours, the number of labeled cells equalized and there was no significant difference between the groups (*P *= 0.81). In the Lin- progenitor cell transfusion model (B), there was no significant difference in the proportion of labeled cells between the control and pneumonia groups (10.1 ± 1.5% vs. 12.9 ± 1.2%, *P *= 0.19) at 2 hours. After 24 hours, the ratio of donor cells in the pneumonia group increased significantly as compared to control (*P *< 0.001) and the previous timepoint (*P *= 0.006). Data is shown as mean ± SE; n = 6 (control group for each timepoint) and n = 7 (pneumonia group for each timepoint). **P < 0.01.

The proportion of QD-labeled donor cells (fraction of total injected cells) that sequestered and homed to the BM is shown in Figure 3. In the whole BMC transfusion model (Figure 3A), there was a trend towards increased homing of QD-labeled donor cells into the BM in the pneumonia animals as compared to control (7.7 ± 0.6% vs. 6.1 ± 0.5%, *P *= 0.09) at 2 hours post-transfusion. At 24 hours, the proportion of QD-labeled cells in both control and pneumonia animals were not different (6.5 ± 0.3% vs. 6.7 ± 0.6%, *P *= 0.81). There was also no significant difference between 2 and 24 hours post-transfusion in both groups.

In the Lin- PC transfusion model (Figure 3B), the proportion of QD-labeled donor cells that were sequestered in the BM at 2 hours post-transfusion was similar between the control and pneumonia groups (10.1 ± 1.5% vs. 12.9 ± 1.2%, *P *= 0.19). After 24 hours, the ratio of labeled cells homing to the BM in the pneumonia group increased significantly (18.5 ± 0.8%, *P *= 0.006) as compared to the 2 hour timepoint and the fraction of cells was also significantly higher as compared to control (18.5 ± 0.8% vs. 9.5 ± 0.4%, *P *< 0.001).

### Sequestration of donor cells in recipient lungs

**Figure 4:**
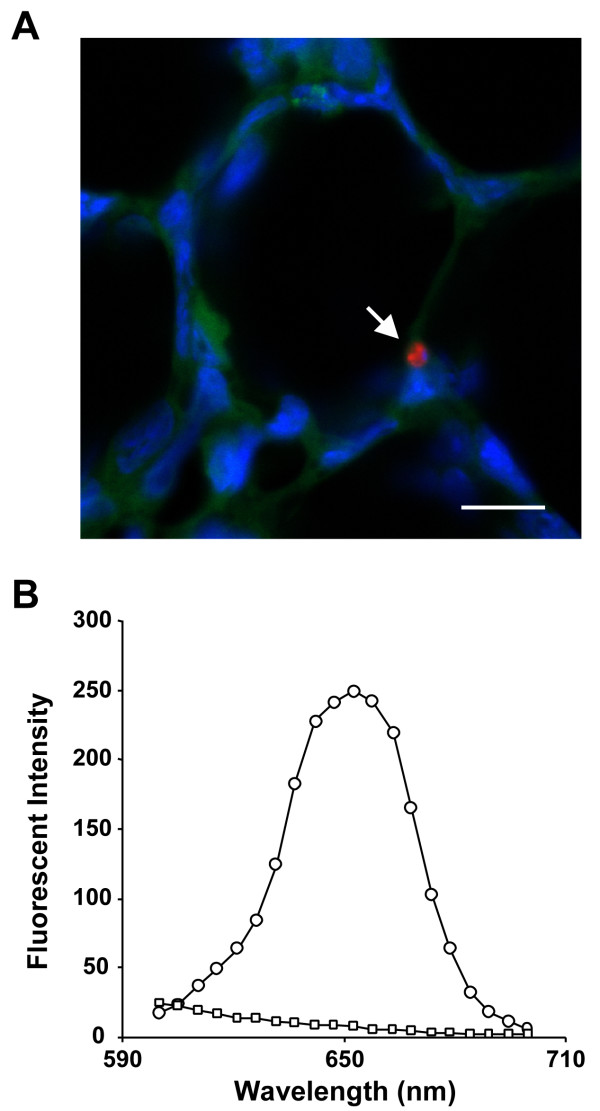
**Labeled donor cells in recipient lung**. A representative image of a QD-labeled donor cell (arrow) as detected in recipient's lung tissue under confocal microscopy (A). Blue denotes nuclei stained with Hoechst 33342, green is autofluorescence from lung tissue, and red is emission signal from quantum dots. The graph (B) shows the wavelength and fluorescence level of QDs on the labeled cell (circle) and lung tissue (square). The sharp peak at 655 nm indicates that the emission from QDs is very distinct from the autofluorescence of lung tissue. Scale bar is 10 μm.

Figure 4 shows a QD-labeled donor cell in recipient lung tissue as viewed using confocal microscopy with an emission signal peak of 650 nm.

**Figure 5:**
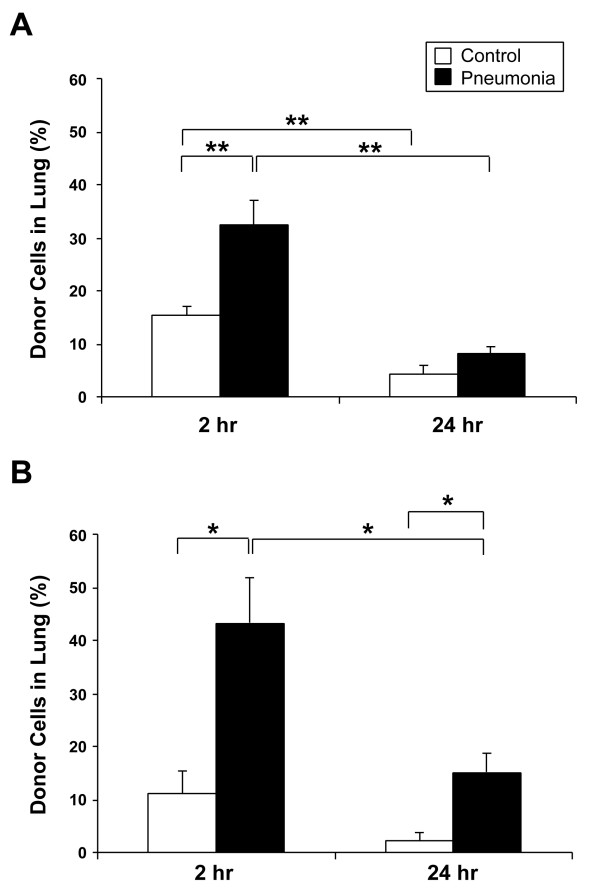
**Frequency of labeled donor cells in lung**. The total number of QD-positive donor cells sequestered in the whole lung was calculated by morphometric analysis. The results are shown as the donor cell proportion of total injected cells that were sequestered in the lung. In the whole BM cell transfusion model (A), significantly more donor cells were sequestered in the pneumonia lungs as compared to control (32.6 ± 4.6% vs. 15.3 ± 1.8%, *P *= 0.007) at 2 hours post-transfusion. After 24 hours, the number of donor cells in lung significantly decreased in both groups, and the difference between the two groups was no longer significant. In the Lin- progenitor cell transfusion model (B), there was a significantly higher number of donor cells sequestered in the pneumonia lungs as compared to control (43.3 ± 8.6% vs. 11.2 ± 3.9%, *P *= 0.03) at 2 hours post-transfusion. After 24 hours, although the percentage of donor cells decreased in both groups, the ratio of donor cells in the pneumonia group was still significantly higher than control (15.1 ± 3.6% vs. 2.4 ± 1.2%, *P *= 0.04). Data is shown as mean ± SE; n = 6 (control group for each timepoint) and n = 7 (pneumonia group for each timepoint). *P < 0.05, **P < 0.01.

Figure 5 shows the proportion of labeled donor cells, expressed as a fraction of total injected cells, which were sequestered in the lung. In the whole BMC transfusion model (Figure 5A), significantly more donor cells were sequestered in the pneumonia lungs as compared to control at 2 hours post-transfusion (32.6 ± 4.6% vs. 15.3 ± 1.8%, *P *= 0.007). By 24 hours, the number of labeled cells remaining in the lung significantly decreased in controls (4.1 ± 1.7%, *P *= 0.001) and in the pneumonia group (8.3 ± 1.1%, *P *< 0.001) as compared to the previous timepoint with no significant difference between the control and pneumonia groups.

In the Lin- PC transfusion model (Figure 5B), significantly more donor cells were sequestered in the pneumonia lungs as compared to control at 2 hours post-transfusion (43.3 ± 8.6% vs. 11.2 ± 3.9%, *P *= 0.03). After 24 hours, as compared to 2 hours post-transfusion, the donor cells remaining in the lung decreased in both the control (2.4 ± 1.2%) and the pneumonia groups (15.1 ± 3.6%) although the decrease was significant only in the latter (*P *= 0.04). The percentage of donor cells remaining in the lungs of the pneumonia group was still significantly higher than the control group (*P *= 0.04) at this timepoint.

### Migration of donor cells into alveolar air space in pneumonia lungs

**Figure 6:**
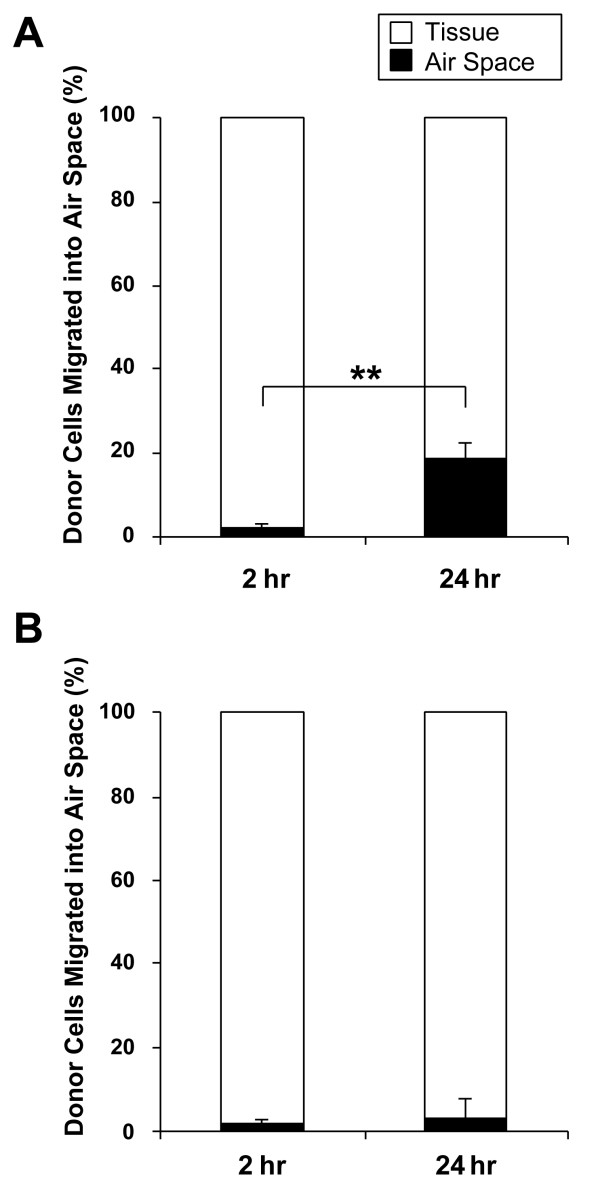
**Migration of labeled donor cells into alveolar air space**. In pneumonia lungs, the ratio of cells that migrated into alveolar air space was measured. The results are shown as the percentage of donor cells that migrated into air space out of the total number of donor cells sequestered in pneumonia lung. In the whole BM cell transfusion model (A), a significantly higher number of donor cells migrated into the air space of pneumonia lungs at 24 hours post-transfusion as compared to 2 hours (18.7 ± 3.7% vs. 2.2 ± 1.2%, *P *= 0.001). In the Lin- progenitor cell transfusion model (B), no significant difference was observed between the 2 and 24 hour timepoints (1.7 ± 1.1% vs. 3.1 ± 4.3%, *P *= 0.60). Data is shown as mean ± SE; n = 7. **P < 0.01.

We next investigated the localization of the labeled donor cells in the pneumonia lungs and detected migration of these cells into alveolar air spaces. In the whole BMC transfusion model (Figure 6A), the percentage of donor cells that migrated into the alveolar air space in pneumonia lungs was 2.2 ± 1.2% and 18.7 ± 3.7% at 2 and 24 hours post-transfusion, respectively, and the difference between the two timepoints was significant (*P *= 0.001).

In the Lin- PC transfusion model (Figure 6B), 1.7 ± 1.1% and 3.1 ± 4.3% of donor cells were found in alveolar air space of pneumonia lungs at 2 and 24 hours, respectively. No significant difference was observed between the two timepoints (*P *= 0.60).

## Discussion

In this study, we demonstrated that both whole BMCs and BM-derived Lin- PCs were sequestered (2 hours) in the lung while the Lin- PCs were preferentially retained (24 hours) there during pneumococcal pneumonia. Furthermore, these progenitor cells also remained in the lung tissues and rarely migrated into the alveolar air spaces, suggesting that BM-derived progenitor cells sequester and home to lung tissues. Several studies have shown increased circulating progenitor cells in lung injury models [26-28], as well as the participation of progenitor cells in the repair response of the lung [18-21]. We propose that during infectious inflammation of the lung, these BM-derived progenitor cells could contribute to either lung inflammation and/or repair following lung infection.

We used QDs to label and trace donor cells in recipients. QDs are highly luminescent semiconductor nanocrystals (CdSe/ZnS-core/shell) and their surface chemistry is modified with peptides so that they can be delivered into cytoplasm of live cells by endocytosis. The advantages of QDs over conventional organic fluorophores are their high levels of brightness, resistance to photobleaching, wide range of excitement wavelengths, and tunable fluorescent wavelengths depending on the size of the particles [29,30]. QDs are now widely used for cellular imaging and many studies have shown the ability to trace diverse types of cells following cell proliferation and differentiation for up to a week without any effects on cell activation or cell function [22,23,31]. We used whole BMCs and BM-derived Lin- PCs and the labeling efficiency of QDs was approximately 80% for both cell types (Figure 1C). Although QDs may be cytotoxic to live cells due to their reactive metal core [23], we used low QD concentrations that have been shown to be noncytotoxic and have no impact on cell function, such as cell proliferation and differentiation [23]. Due to the brightness and high emission signals of QDs, resistance to photobleaching and retention in labeled cells, we were able to clearly identify labeled cells in the blood, BM and the lung using a combination of flow cytometry and confocal microscopy.

Plett and colleagues have shown that BM-derived progenitor cells are rapidly cleared from the circulation after transfusion [32]. In our study 92.3% of the transfused Lin- PCs were cleared from recipient's circulation within 2 hours after the injection in control group. Interestingly, this clearance from the circulation occurred much more rapidly than those shown for transfusion of labeled neutrophils [33] or labeled monocytes [5]. Cell size and deformability have been shown to be important in determining removal of infused cells from the circulation [34]. Immature BM-derived granulocytes are larger and less deformable than mature granulocytes [35], and we hypothesize that cell immaturity is responsible for the rapid clearance of Lin- PCs from the circulation.

In the pneumonia model, however, the clearance of Lin- PCs from the circulation at 2 hours following infusion decreased (Figure 2). Interestingly, these findings are different from the results we have shown previously on neutrophils and monocytes. In bacterial infection, neutrophils and monocytes are more rapidly cleared from the circulation to be sequestered into the infected lungs [6,36]. A unique and independent mechanism might exist for the mobilization and sequestration of progenitor cells. Increased number of circulating progenitor cells has been shown in tissue injury animal models [26,27] as well as in clinical settings including burn, post-cardiac surgery [37], and pneumonia [28] patients. These findings suggest that progenitor cells acquire the capability to remain in the circulation during systemic inflammation for subsequent sequestration into target organs upon demand. The reason for this prolonged stay in the circulation is unclear and needs further investigation. It could be that these cells do not respond to chemo-attractants in the acute inflammatory milieu but are recruited later when cells for resolution and repair are required. Alternatively, these cells may remain in the less hostile intravascular milieu to proliferate and mature, to home and migrate into the inflammatory site at a stage when the acute inflammatory response has been dampened.

Several studies have reported that BM-derived progenitor cells accumulate in lung tissue and have the ability to replace damaged cells following injury [18-21]. We show here that significant sequestration of transfused Lin- PCs occurred in pneumonic lungs at 2 hours as compared to control lungs, suggesting preferential sequestration of these cells in inflamed lung tissues. To determine homing of BM-derived cells to inflamed lung tissues, we investigated the localization of these cells at 24 hours after transfusion. Using purified Lin- PCs, approximately 15.1% of labeled cells were still in the lung at 24 hours while 2.1% remained in the circulation, showing a 7 times enrichment of Lin- PCs in the pneumonia lungs. Few of these Lin- PCs migrated into the airspaces (approximately 3.1% of all the cells remaining in the lung), therefore approximately 14.6% of the initially infused Lin- PCs homed to lung tissues and were potentially available for lung tissue regeneration and repair. These findings showed firstly that very few progenitor cells migrate into the airspaces to participate in airspace inflammation but the majority of cells remain in the lung tissues where they have the potential, via proliferation and differentiation, to participate in lung structural repair. Secondly, a significant fraction of BM-derived progenitor cells homed to the injured lung as compared to the control lung. If this fraction of progenitor cells that home to damaged lung regions remain constant (as shown in the BM homing study by Szilvassy and colleagues [38]), increasing the number of infused progenitor cells will increase the number of these cells that home to injured lung tissues and become available for tissue regeneration and repair.

Interestingly, in the present study, approximately 10–13% of injected Lin- PCs, both in the control and pneumonia groups, returned to the marrow at 2 hours, which supports previous work by other authors [32,38]. However, more Lin-PCs homed back to the marrow at 24 hours in pneumonia group (Figure 3B). This could represent the marrow's ability to recruit progenitors from the circulation pool during infection, in an attempt to produce additional inflammatory cells demanded from the BM by the infection.

## Conclusion

Our study showed that during pneumonia, BM-derived lineage negative progenitor cells remain in the intravascular space for a prolonged time, preferentially sequester in the inflamed lung tissues and are enriched in the lung over a short period of time (24 hours). These cells may play an important role in inflammatory responses against lung infection as well as contributing in tissue repair processes following the infection. Further studies will be required to elucidate the mechanism of behavior of these progenitor cells and to determine the phenotypic characteristics of the cell type responsible for the tissue reparation. Together these concepts may pave the way for future novel cell-based therapy for lung tissue repair following injury.

## Competing interests

The author(s) declare that they have no competing interests.

## Authors' contributions

HS carried out the experiments, performed data analyses and drafted the manuscript. JH participated in interpretation and critical review of data as well as the revision of the manuscript for important intellectual content. SvE conceived the study and made substantial contributions to conception, design and drafting of the manuscript. All authors read and approved the final manuscript.
